# Gestational Weight Gain and Peripartum Cardiomyopathy in a Twin Pregnancy

**DOI:** 10.1155/2015/317146

**Published:** 2015-06-28

**Authors:** Hiroko Matsumiya, Naotaka Saito, Hisanori Minakami, Soromon Kataoka

**Affiliations:** ^1^Department of Obstetrics and Gynecology, Hakodate Central General Hospital, Hakodate 040-8585, Japan; ^2^Department of Cardiovascular Medicine, Hakodate Central General Hospital, Hakodate 040-8585, Japan; ^3^Department of Obstetrics, Hokkaido University Graduate School of Medicine, Sapporo 001-0014, Japan

## Abstract

Preeclamptic twin pregnancy with larger gestational weight gain (GWG) is suggested to have a higher risk of peripartum cardiomyopathy (PPCM). This was true in a 5-year experience at a single center. A primiparous woman with twins and prepregnancy weight of 51.0 kg exhibited hypertension at gestational week (GW) 32^−6/7^ and GWG of 18.3 kg (6.0 kg and 2.9 kg during the last four weeks and one week of gestation, resp.) concomitant with generalized edema, gave birth at GW 34^−4/7^, developed proteinuria, cough, and dyspnea postpartum, and was diagnosed with preeclampsia and PPCM showing left ventricular ejection fraction of 34% and plasma BNP level of 1530 pg/mL. This was the only case of PPCM among 101 (12 with preeclampsia) and 3266 women with twin and singleton pregnancies, respectively. Thus, PPCM occurred significantly more often in women with preeclamptic twin pregnancies than in women with singleton pregnancies (8.3% [1/12] versus 0.0% [0/3266], *P* = 0.0355). This patient showed the greatest weight gain of 6.0 kg during the last four weeks of gestation and the greatest weight loss of 19.2 kg during one month postpartum among 90 women with twin deliveries at GW ≥ 32.

## 1. Introduction

Peripartum cardiomyopathy (PPCM) is an idiopathic cardiomyopathy presenting with heart failure (HF) in which dyspnea, orthopnea, tachycardia, and peripheral edema can be symptoms [1, 2]. The PPCM is defined by the European Society of Cardiology as follows: “idiopathic cardiomyopathy presenting with HF secondary to left ventricular (LV) systolic dysfunction toward the end of pregnancy or in the months following delivery, where no other cause of HF is found. It is a diagnosis of exclusion. The LV may not be dilated but the ejection fraction (EF) is nearly always reduced below 45%” [2]. The majority of patients diagnosed during pregnancy present in the third trimester, with a few in the second trimester [3, 4].

Clinical symptoms associated with PPCM include orthopnea, dyspnea, and edema [5]. However, as edema is common in healthy pregnant women [6] and as PPCM is a rare complication occurring in 1 per 2000–4000 women [5, 7–9], diagnosis of PPCM is often delayed resulting in major adverse events, such as death or heart transplantation [10]. In epidemiological studies, twin pregnancy and hypertensive disorder in pregnancy are consistent and prominent risk factors for PPCM [1, 3–5, 7, 10–13], accounting for 7%–15% and 15%–68% of all PPCM cases, respectively [4, 11]. Recent case reports suggested an association between greater gestational weight gain (GWG) and the risk of developing PPCM [14, 15]. These reports prompted us to review our previous PPCM case occurring in a preeclamptic woman with twin pregnancy. This case highlighted that risk of PPCM is indeed higher in preeclamptic twin pregnancy with greater GWG. Here, we present the details of this case emphasizing the need for attention to changes in maternal weight in the late stage of twin pregnancy complicated with hypertension. Changes in maternal weights in 90 other women with twin pregnancies were also reported. The Hakodate Central General Hospital (HCGH) Institutional Review Board approved this study and patient gave signed informed consent.

## 2. Case Presentation

A 37-year-old nulliparous Japanese woman (height, 1.56 m; prepregnancy body weight, 51.0 kg) with dichorionic diamniotic twin pregnancy developed hypertension (144/77 mmHg) at gestational week (GW) 32^−6/7^. No episodes suggestive of infection, such as malaise and/or febrile conditions, were present in this patient. Blood pressure (BP) remained stable below 160/95 mmHg in the absence of antihypertensives and she developed neither significant proteinuria (protein loss in urine, 126 mg/day at GW 34^−3/7^), thrombocytopenia < 130 × 10^9^/L, renal dysfunction monitored by serum creatinine, nor clinical symptoms, such as dyspnea. However, elevation of serum AST/ALT from 20/12 to 43/24 IU/L and an increase in maternal weight by 2.9 kg (from 66.4 kg to 69.3 kg, GWG of 18.3 kg) in the last week of gestation concomitant with generalized edema prompted us to perform caesarean section at GW 34^−4/7^. Two otherwise healthy premature male/male twins were born weighing 2003 g and 2059 g with 1- and 5-minute Apgar scores of 8 and 9 and 9 and 9, respectively. Her BP remained below 165/115 mmHg in the absence of antihypertensives postpartum, and she was diagnosed with preeclampsia and PPCM after exhibiting significant proteinuria of 460 mg/day on postpartum day (PPD) 3, orthopnea, cough, and dyspnea with cardiothoracic dimension ratio of 55% and left ventricular ejection fraction (LVEF) of 34% on PPD 5, and plasma brain-type natriuretic peptide level of 1530 pg/mL on PPD 8. She showed body weight loss of 19.2 kg (from 69.3 kg soon before delivery to 50.1 kg) by PPD 29. Her LVEF improved to 44% and 66% on PPD 28 and postpartum month 9, respectively.

## 3. Medical Chart Review

**Figure 1:**
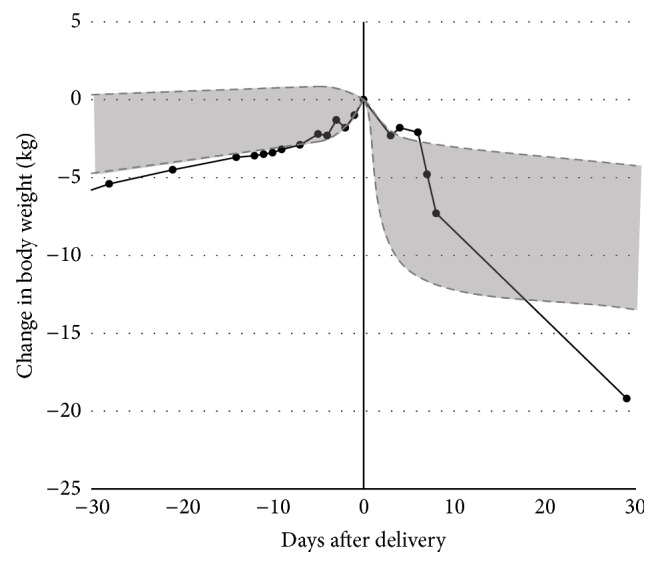
Changes in body weight in a PPCM woman with preeclamptic twin pregnancy. Shaded area indicates the 5th–95th percentile range for 90 women giving birth to twins at gestational week ≥ 32 at Hakodate Central General Hospital. The solid line indicates the present case.

This was the only patient diagnosed with PPCM among 3367 women (3266 with singletons and 101 with twins) giving birth on and after GW ≥ 22 at the HCGH over the 5 years from January 2010 to December 2014. Preeclampsia developed in 112 (3.4%) and 12 (12%) women with singleton and twin pregnancies, respectively. Thus, PPCM occurred significantly more often in women with preeclamptic twin pregnancies than in women with singleton pregnancies (8.3% [1/12] versus 0.0% [0/3266], *P* = 0.0355, Fisher's exact test). Medical charts of 90 women giving birth to twins at GW ≥ 32 at the HCGH were reviewed. Eleven of the 90 women had preeclampsia. The height (median, 5th–95th percentile) was 158 (150–166.6) cm, prepregnancy body mass index was 21.0 (17.4–28.3) kg/m^2^, and GWG was 11.5 (4.8–21.2) kg. Although the patient's GWG of 18.3 kg was not particularly high, corresponding to 87.5th percentile, both the weight gain of 6.0 kg in the last four weeks of pregnancy and weight loss of 19.2 kg during one month postpartum were greatest among the 90 women (Figure 1), suggesting excess water retention in the last month of gestation in this patient. For 79 normotensive women versus 11 preeclamptic women, median GWG was 11.2 versus 13.5 kg (*P* = 0.4114), weight gain during the last four weeks was 2.2 versus 3.6 kg (*P* = 0.8561), and weight gain during the last one week was 0.6 versus 0.75 kg (*P* = 0.9494), respectively.

## 4. Discussion

The present case as well as two previous cases of PPCM reported in the literature [14, 15] exhibited greater changes in body weight during pregnancy. The GWG was 18.3 kg, weight gain in the last four weeks of gestation was 6.0 kg, and weight loss in one month postpartum was 19.2 kg in the present case with delivery at GW 34. In a woman with singleton delivery at GW 34, weight gain in the last three weeks of gestation was 11.6 kg and weight loss in one month postpartum was 24 kg [14]. In another woman with twin delivery at GW 32, GWG and weight loss in one month were 25.5 kg and 28.5 kg, respectively [15]. The GWG, weight gain in the late stage of gestation, and weight loss in one month postpartum of the three women appeared to be greater than those of the general population.

In a study on GWG of Japanese women with delivery at GW ≥ 22 [16], GWG of >20 kg occurred in only 1,404 (1.1%) of 128,838 women with singleton pregnancies and 178 (3.2%) of 5,573 women with twin pregnancies [16]. Among women with delivery at GW 33–36, the mean GWG was 7.9 ± 4.2 kg for 10,136 women with singleton pregnancies and 10.5 ± 4.7 kg for 2,389 women with twin pregnancies [16]. In another study examining 272 healthy Japanese women giving birth to a singleton at term [17], the median and 97.5th percentile values for weight loss during one month postpartum were 7.5 kg and 13.1 kg, respectively [17]. Of the 90 women giving birth to twins at GW ≥ 32 at the HCGH over the past 5 years, none except the present case showed 6.0 kg or more of weight gain during the last four weeks of gestation and weight loss of more than 19 kg during one month postpartum. Thus, three PPCM women, including the present case and two reported in the literature [14, 15], indeed exhibited greater GWG, especially during the late stage of pregnancy, and greater weight loss during one month postpartum compared with the general population.

Umazume et al. suggested that preeclamptic twin pregnancy is associated with higher risk of PPCM [15]. This was true at the HCGH. The GWG is greater in twin than singleton pregnancies [16]. The greater GWG is associated with the development of preeclampsia [18]. In epidemiological studies, multifetal pregnancy and hypertensive disorders are consistent and prominent risk factors for PPCM, accounting for 7%–15% and 15%–68% of all PPCM cases, respectively [1, 3–5, 7, 10–13]. In a study of 102 Japanese women with PPCM [11], 15 and 42 were complicated with multifetal pregnancy and hypertensive disorders, accounting for 15% and 41% of all PPCM cases, respectively. As multifetal pregnancies account for approximately 1.0% of all pregnancies in Japan [19] and hypertension occurs in approximately 5% of all general pregnant Japanese women [20], these results indicated that women with multifetal pregnancies and hypertensive disorders were at approximately 15- and 8-fold higher risk of developing PPCM, respectively, compared with the general population. Although not verified in these studies [1, 3–5, 7, 10–13], it was speculated that GWG, especially weight gain in the late stage of pregnancy, may have been greater in PPCM women than in the general population.

“Weight gain during the last month of pregnancy” was incorporated into a self-test scoring system developed by Fett [21] for early diagnosis of HF. One of the authors (HM) retrospectively assessed the present patient's condition immediately before diagnosis of PPCM using this test—the patient scored 10 (2 on orthopnea, 2 on dyspnea, 2 on unexplained cough, 2 on swelling, 2 on weight gain, and 0 on palpitation), consistent with the results of the study reported by Fett [21] in which the scores for 47 PPCM patients versus 10 control mothers (mean, range) were 8.93 (5–12) versus 1.5 (0–3), respectively.

An imbalance between an angiogenic agent (vascular endothelial growth factor [VEGF]) and an antiangiogenic factor (soluble fms-like tyrosine kinase-1 [sFlt-1]) is seen in women with preeclampsia [22–24], twin pregnancy [24], and PPCM [24], and an antiangiogenic environment is seen in these three conditions with elevated levels of sFlt-1 [22–24]. The elevated sFlt-1 is associated with widespread endothelial dysfunction [23] that may increase vascular permeability [25]. The increased vascular permeability allows excess water retention in the interstitial space forming edema. This may be associated with greater GWG in the last month of pregnancy in this case as well as two previous cases with PPCM.

In conclusion, the present case as well as two previous cases reported in the literature [14, 15] emphasized the need for attention to changes in body weight of pregnant women, especially in preeclamptic twin pregnancies, with respect to the risk of PPCM.
